# Implicit and Explicit Attention to Pictures and Words: An fMRI-Study of Concurrent Emotional Stimulus Processing

**DOI:** 10.3389/fpsyg.2015.01861

**Published:** 2015-12-18

**Authors:** Tobias Flaisch, Martin Imhof, Ralf Schmälzle, Klaus-Ulrich Wentz, Bernd Ibach, Harald T. Schupp

**Affiliations:** ^1^Department of Psychology, University of KonstanzKonstanz, Germany; ^2^Department of Radiology, Kantonsspital MünsterlingenMünsterlingen, Switzerland; ^3^Department of Psychiatry, Psychiatrische Dienste ThurgauMünsterlingen, Switzerland

**Keywords:** emotion, language, pictures, attention, perception, fMRI

## Abstract

The present study utilized functional magnetic resonance imaging (fMRI) to examine the neural processing of concurrently presented emotional stimuli under varying explicit and implicit attention demands. Specifically, in separate trials, participants indicated the category of either pictures or words. The words were placed over the center of the pictures and the picture-word compound-stimuli were presented for 1500 ms in a rapid event-related design. The results reveal pronounced main effects of task and emotion: the picture categorization task prompted strong activations in visual, parietal, temporal, frontal, and subcortical regions; the word categorization task evoked increased activation only in left extrastriate cortex. Furthermore, beyond replicating key findings regarding emotional picture and word processing, the results point to a dissociation of semantic-affective and sensory-perceptual processes for words: while emotional words engaged semantic-affective networks of the left hemisphere regardless of task, the increased activity in left extrastriate cortex associated with explicitly attending to words was diminished when the word was overlaid over an erotic image. Finally, we observed a significant interaction between Picture Category and Task within dorsal visual-associative regions, inferior parietal, and dorsolateral, and medial prefrontal cortices: during the word categorization task, activation was increased in these regions when the words were overlaid over erotic as compared to romantic pictures. During the picture categorization task, activity in these areas was relatively decreased when categorizing erotic as compared to romantic pictures. Thus, the emotional intensity of the pictures strongly affected brain regions devoted to the control of task-related word or picture processing. These findings are discussed with respect to the interplay of obligatory stimulus processing with task-related attentional control mechanisms.

## Introduction

Multiple processes determine the regulation of selective attention processes. On the one hand, selective attention can be regulated voluntarily (i.e., “explicitly”) if attention is focused on goal-relevant stimuli in the environment. On the other hand, inherent features of a stimulus may also regulate attention processes (i.e., “implicitly”) such as when novel stimuli appear suddenly in the environment or when pictures grab attention due to the emotional significance conveyed by the image^1^. A large array of studies was conducted to examine the interaction among implicit and explicit processes in the regulation of selective attention processes. Interaction effects were detailed with respect to implicit emotion and explicit goal relevance in conditions of cooperation and competition for processing resources, as well as in conditions of implicit emotion significance in different sensory modalities. To extend these lines of research, the present study investigated effects of both cooperation and competition among emotionally arousing and neutral stimuli by directing the task focus to either words or the scene of the image presented concurrently in a compound stimulus.

### Selective attention: implicit and explicit processes

Explicitly directed attention toward visual features, objects, and higher-order semantic categories revealed accentuated activations in occipital and inferior temporal cortical regions preferentially engaged by specific stimulus attributes such as color, stimulus orientation, or object category (Kastner and Ungerleider, 2000; Vuilleumier, 2005; Jehee et al., 2011). Additionally, the activity in these regions is also modulated by explicit spatial attention. Specifically, directing attention toward a lateralized stimulus in either visual hemifield enhances activity in corresponding areas contralaterally to the location of the stimulus (Heinze et al., 1994; Mangun et al., 1998; Kastner and Ungerleider, 2000). Thus, explicit attention toward visual stimuli regulates selective attention processes in sensory-perceptual brain regions.

A similar pattern of findings was seen in studies examining the implicit regulation of attention processes by varying emotional arousal of the stimuli. Specifically, a large array of studies consistently demonstrated that the processing of emotionally arousing (pleasant and unpleasant) as compared to non-emotional picture stimuli leads to increased activations in extended regions of the visual system including the extrastriate visual cortex and widespread regions of the inferior temporal cortex (Lang et al., 1998; Junghöfer et al., 2005; Sabatinelli et al., 2005; Flaisch et al., 2009). Of note, in these studies those effects are also reliably observed when participants view pictures passively and when the task does not require them to actively process the stimulus' emotional connotation. In sum, explicit task-relevancy, as well as the emotional significance of pictures regulate attention processes in brain regions devoted to visual stimulus processing.

Beyond pictures, there is also robust evidence for the preferential processing of emotional words (reviewed in Citron, 2012). Specifically, emotional (positive and negative) as compared to neutral words (i.e., nouns and adjectives) elicited increased activations in the inferior and middle frontal gyrus, middle temporal gyrus, dorso-medial prefrontal cortex, and inferior parietal lobe (Cato et al., 2004; Kensinger and Schacter, 2006; Herbert et al., 2009; Hoffmann et al., 2015). Furthermore, these effects are often obtained most reliably in left-hemispheric regions (Kotz and Paulmann, 2011). Thus, emotional words regulate attention processes in a brain network devoted to semantic processing with the left-hemispheric focus being consistent with a large array of studies examining non-emotional language processing (Price, 2012). As with pictures, visually presented emotional words also engage extrastriate visual areas (Kensinger and Schacter, 2006). In one instance this occurred exclusively in the left hemisphere (Herbert et al., 2009), suggesting an overlap in neural regions for the visual processing of emotional pictures and words.

However, the mechanism of preferential stimulus processing seems to be at least partially different for implicit emotional and explicit task-related attention processes. In many studies, the amplified processing of emotional pictures is accompanied by activation increases in limbic and para-limbic regions, i.e., the amygdala, orbitofrontal cortex, cingulate gyrus, and dorso-medial prefrontal cortical regions (Junghöfer et al., 2005; Sabatinelli et al., 2011; Lindquist et al., 2012). Similarly, limbic structures also respond to the emotionality of words, most prominently the amygdala (Hamann and Mao, 2002; Cato et al., 2004; Kensinger and Schacter, 2006; Herbert et al., 2009; Kanske and Kotz, 2011; Straube et al., 2011; Hoffmann et al., 2015). While the specific outcome of an individual study may vary, possibly due to differences in experimental design, used stimuli, or technical constraints, recent meta-analyses largely confirmed the involvement of these regions (Sabatinelli et al., 2011; Lindquist et al., 2012). On the other hand, explicit attention studies usually reveal the activation of distinct neural structures which are thought to regulate selective attention processes. Specifically, the regulation of attention has been associated with activity in frontal cortical regions, including frontal and supplementary eye fields as well as the dorso-lateral prefrontal cortex accompanied by regions of the superior and inferior parietal lobe (Desimone and Duncan, 1995; Kastner and Ungerleider, 2000; Corbetta et al., 2008). In sum, while implicit emotional and explicit task-related attention processes share common neural substrates such as enhanced sensory-perceptual processing, they are also characterized by distinct activations in limbic brain areas implicated in emotion processing and prefrontal regions associated with the volitional regulation of selective attention, respectively.

### Selective attention: the interaction among implicit and explicit processes

Studying the interaction of implicit emotional and explicit attention processes was spurred by examining the hypothesis that emotion processing occurs automatically. In a first study, Vuilleumier et al. (2001) presented multiple stimuli, i.e., faces (fearful and neutral) and houses aligned vertically and horizontally, and directed the participants' explicit attentional focus either toward the faces or the houses by asking them to decide whether the respective stimulus dimension showed the same pictures or not. Supporting the notion of automaticity, the selective processing of fearful and neutral faces was maintained in the amygdala and fusiform cortex even when the focus of attention was on the house stimuli. There were also neural regions responsive to fearful faces only when the stimuli were the focus of attention, e.g., anterior cingulate and orbitofrontal cortex. Thus, while selective emotion processing in some brain regions appears to depend on explicit task-focus, others seem to respond to stimulus emotionality automatically, i.e., even if they are processed outside the explicit focus of attention. However, the notion of automaticity has been challenged by subsequent studies. For instance, Pessoa et al. (2002) reported emotionally enhanced activity in the amygdala and visual cortex only if the emotional faces were actively attended. Since then, numerous studies have confirmed the finding that implicit attention to emotion competes with explicit attentional demands not only in the amygdala but also in other brain regions, consequently decreasing preferential emotion processing under conditions of heightened task-load and/or distraction (Blair et al., 2007; Hsu and Pessoa, 2007; Mitchell et al., 2007; Van Dillen et al., 2009; McRae et al., 2010; Yates et al., 2010; Kanske and Kotz, 2011).

In addition to studying the interaction of implicit emotion and explicit attention processes, multisensory studies enable examining the interaction of multiple implicit processes by concurrently presenting emotional stimuli in different sensory modalities (for recent reviews see Klasen et al., 2012; Gerdes et al., 2014). In according studies, participants view e.g., emotional facial expressions while listening at the same time to human voices with emotionally modulated prosody. The findings demonstrate the concurrent preferential processing of emotional stimuli in different modalities. Specifically, visual emotional stimuli elicited increased activity in primary and associative visual cortical regions and, simultaneously, auditory emotional stimuli enhanced activity in primary and higher-order auditory cortices (e.g., Ethofer et al., 2006). This finding suggests that the brain is able to process the concurrent call for preferential processing in parallel when the different sources of emotional significance demand resources from different processing channels. Accordingly, this is consistent with the notion put forward by Lavie (2005) maintaining that competition effects are primarily a function of competition for shared processing resources. On the other hand, this also implies that competition effects should be more pronounced when several concurrent sources of implicit emotional significance within the same sensory modality demand shared processing resources.

### The present study

The present study was designed to further detail the emotion-attention relationship by exploring how the brain processes concurrently presented visual emotional stimuli under varying explicit and implicit attention demands. Toward this end, the different lines of research, i.e., explicit attention and preferential processing of emotional words and pictures were brought together in the present study with the intent to capitalize on the finding that the preferential processing of emotional pictures and words is associated both with shared, as well as distinct brain regions. Specifically, while implicit emotional attention conveyed by either stimulus class is associated with enhanced perceptual processing, emotional words in particular are characterized by stimulus-specific activation increases in semantic brain regions associated with word processing. This allowed us to assess effects of implicit and explicit attention on stimulus-specific and shared brain regions by presenting the two stimulus classes simultaneously. A task varying between trials manipulated the focus of attention by asking participants to indicate either the pre-defined category of the pictures as “erotic” vs. “everyday,” or of the words as “positive” or “neutral.” Consequently, when attention was directed toward one class of stimuli, i.e., picture or word, the other stimuli were task-irrelevant. The main goals of the present study were to assess neural structures implicated in regulating explicit attention toward pictures and words and to examine the interaction of attention with emotional stimulus significance. A first set of hypotheses regarded main effects of emotional intensity and explicit task instruction. Based on previous findings on picture and word processing, it was predicted that emotionally arousing pictures and words are preferentially processed as compared to control stimuli in regions of the extended visual cortex for pictures and (left-hemispheric) regions of the semantic network for words. In the present study design, simple main effects of the task indicate the net effect between the attention focus toward and away from either the picture or word stimuli. The phrase “a picture is worth a thousand words” indicates that pictures are more salient than words. Accordingly, it was predicted that the demand of attention regulation is most pronounced for the picture categorization task. In addition, the overlap of task activations with regions sensitive to the emotional significance of stimuli would suggest that such effects are associated with selective attention, *per se*, rather than reflecting attention control regions which should only be observed as a function of the task manipulation. Finally, the need for attention control is presumed to vary for emotional and neutral stimuli serving as target and distracter stimuli. Specifically, diverting attention away from erotic stimuli seems most challenging, leading to an interaction of Picture Category by Task most likely observed in pre-frontal and parietal regions associated with attention regulation and showing greater activation for word categorization trials presented over task-irrelevant erotic pictures.

## Materials and methods

### Participants

Thirty-one volunteers (18 females; 1 left-handed) between 18 and 34 years of age (*M* = 21.8) with normal or corrected-to-normal vision participated in the study. Behavioral data for two participants were lost due to technical problems. Thus, data from 29 participants entered behavioral analysis. All participants were native German speakers. They were recruited at the University of Konstanz and received either course credits or €8 per hour. All participants provided informed consent to the study protocol, which was approved by the ethical review board of the University of Konstanz. All participants were healthy at the time of measurement and reported no history of neurological or psychiatric disorders.

### Stimulus materials, tasks, and experimental procedure

Word stimuli were selected from the Berlin Affective Word List Reloaded (BAWL-R; Võ et al., 2009) and included 22 emotionally positive and 22 neutral German nouns^2^ referring to different categories of human experience. According to normative ratings, the categories differed in terms of valence (positive: *M* = 8.1, SD = 0.32; neutral = 5.0, SD = 0.32; *p* < 0.001) as well as arousal (positive: *M* = 5.7, SD = 1.13; neutral: *M* = 3.5, SD = 0.82; *p* < 0.001)^3^. The two word categories were matched for word length (3–6 letters), number of syllables (1–3), imageability, and word frequency (Võ et al., 2009).

Picture selection comprised 22 images of nude couples in erotic poses and 22 images of dressed couples in romantic situations. Previous research provides strong evidence that the activation of visual-associative as well as subcortical limbic structures is driven by the emotional arousal dimension and accentuated for erotic stimuli (Junghöfer et al., 2005; Sabatinelli et al., 2005). The “romantic” control category was selected to promote the comparability of the two picture categories in terms of picture composition and categorical homogeneity. Specifically, pictures did not differ in complexity, color, or number of people i.e., all pictures were black and white and showed heterosexual dyads of socially interacting couples. Subjective ratings collected from an independent sample of 16 participants (8 females) revealed that both picture categories did not differ regarding valence (self-assessment manikin; Bradley and Lang, 1994; erotic: *M* = 5.8, SD = 1.16; romantic: *M* = 6.3, SD = 1.13; ns.), but that erotic images were rated as significantly more arousing (erotic: *M* = 6.3, SD = 0.99; romantic: *M* = 2.7, SD = 1.09; *p* < 0.001).

**Figure 1:**
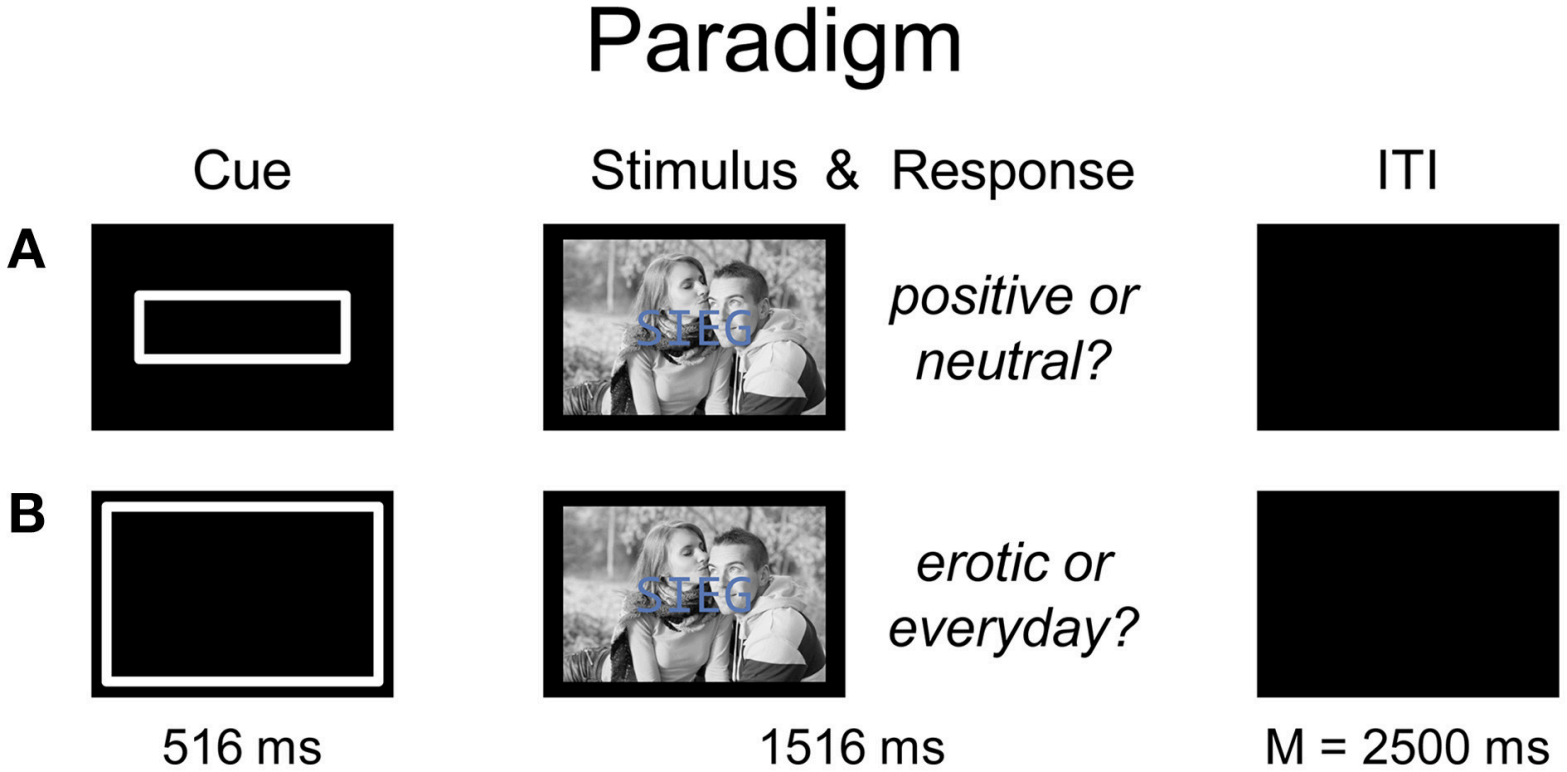
**Illustration of the trial sequence**. A pre-stimulus box cue indicated whether participants should categorize the word **(A)** or the picture **(B)** in the present trial. Then the compound picture-word stimulus was displayed, and the participant responded. During a variable inter-trial-interval, a blank screen was shown before the next pre-stimulus cue was presented. Please note that the photograph used in Figure 1 is shown for exemplary reasons and was not part of the stimulus set [“love” by Richard foster (https://www.flickr.com/photos/93963757@N05/8550837497), used under CC BY-SA 2.0 (https://creativecommons.org/licenses/by-sa/2.0/), decolorized from original].

The compound stimulus was constructed by centrally overlaying the respective word, in gray-blue capital letters and Consolas font, over the respective erotic or romantic pictures (Figure 1). For each participant, the respective pairings of specific words and pictures were randomly assigned for each experimental cell of the *Picture Category*-by-*Word Category* interaction (i.e., erotic-positive, erotic-neutral, romantic-positive, romantic-neutral). This assignment was then kept constant across the *Task* factor, i.e., each participant viewed the same word-picture combinations twice, once under the word and once under the picture categorization instruction, respectively. This resulted in eight experimental cells overall. The stimuli were displayed on a back-projection screen and participants viewed them via a mirror attached to the head-coil. The pictures subtended a vertical visual angle of 16.1° and a horizontal visual angle of 21.5°; the words subtended vertically 3.9° and horizontally between 9.8° (3-letter word) and 19.6° (6-letter word). A white rectangle on a black background served as pre-stimulus response cue and its size was matched to the picture or word stimulus-dimension to signal an upcoming picture or word categorization trial.

To minimize effects of task difficulty and to avoid categorical ambiguity, participants were familiarized with the entire stimulus set and each stimulus' categorical assignment before scanning. Toward this end, participants were shown each exemplar of the two picture and two word categories in separate blocks and the distinct labels for the picture (erotic or everyday) and the word (positive or neutral) categories were introduced. The order of blocks during familiarization was randomized across participants. Afterwards, participants received the instructions and then worked through 12 practice trials for which random stimuli were drawn from the regular stimulus set. The task was to categorize either the background picture or the overlaid word as fast and as accurately as possible. To minimize effects of response conflict, each response alternative was assigned to a specific finger, respectively, and differing verbal descriptions for picture and word categories were deliberately chosen to avoid direct semantic mapping onto each other. Participants responded by pressing the corresponding right and left index and middle fingers, respectively. Hereby, picture category had to be categorized with one, and word category with the other hand, balanced across participants. “Erotic picture” and “positive word” as well as “everyday picture” and “neutral word” were always mapped onto either the index or the middle fingers, which was again balanced across participants.

Each trial began with the presentation of a pre-stimulus cue for 516 ms indicating the stimulus dimension to be categorized, i.e., word or picture, followed by the main compound stimulus for 1516 ms, and a black inter-trial-interval (ITI) whose duration was exponentially distributed with a mean of 2500 ms and a range of 2000–4000 ms (Dale, 1999; Figure 1). The main experiment comprised 352 trials (44 per experimental cell) which were presented consecutively in a single session lasting approximately 29 min. Hereby, order of trials was randomized and the same picture or word could not appear in succession.

### Data acquisition and analysis

Scanning was conducted using a 3-Tesla Siemens Verio MR-System. For functional imaging, a T2^*^-weighted gradient single-shot echo planar imaging (EPI) sequence was acquired. In-plane resolution was 3.0 × 3.0 mm and slice thickness was 3.5 mm (36 axial slices; no gap; FOV = 240 mm; acquisition matrix: 80 × 80 voxels; TE = 30 ms; flip angle = 90°; TR = 2500 ms). In addition, a high-resolution T1-weighted structural scan was obtained for each participant.

Statistical analyses of the functional images were conducted using Statistical Parametric Mapping (SPM8; Wellcome Department of Imaging Neuroscience, University College London, UK; http://www.fil.ion.ucl.ac.uk/spm/software/spm8; Friston et al., 1994). Preprocessing included slice-time correction and realignment without unwarping. Additionally, the functional images were spatially normalized to the standard EPI-template and smoothed with a kernel of FWHM = 8 × 8 × 8 mm. On the fixed-effects level, the data were analyzed in an event-related design comprising eight covariates-of-interest classifying each trial in terms of *Picture Category* (erotic vs. romantic), *Word Category* (positive vs. neutral), and experimental *Task* (picture categorization vs. word categorization). To improve model-fit, additional covariates-of-no-interest were included comprised by the modeled covariates-of-interest's time and dispersion derivatives, six movement parameters obtained during realignment, and one covariate incorporating an overall intercept to the model. A high-pass filter with a cutoff period of 128 s was applied to the data. To avoid a bias of the global signal from the emotionally intense erotic picture category, no global scaling was applied (Junghöfer et al., 2005). BOLD-activity associated with each experimental condition was determined by contrasting each covariate-of-interest with the implicit baseline.

Random-effects analysis was implemented by calculating a flexible-factorial model including the within-subject main effects of *Picture Category* (erotic vs. romantic), *Word Category* (positive vs. neutral), and *Task* (picture categorization vs. word categorization), as well as all possible two-way interactions. Additionally, a subject factor was included in the model to account for between subject variance. Activated voxels were determined by means of bi-directional F-contrasts for interactions and directed T-contrasts for main effects and were considered meaningful if they reached a statistical threshold of *p* < 0.05 (FDR-corrected at voxel level, cluster size *k* > 15). Figures were created using MRIcron software (http://www.mccauslandcenter.sc.edu/mricro/mricron/; Rorden and Brett, 2000) displaying activations in neurological orientation. Coordinates in Tables 1–**5** are reported in MNI space, and the respective labels of their anatomical locations were obtained using the maximum probability tissue atlas from the OASIS-project (http://www.oasis-brains.org/) as provided in SPM12 by Neuromorphometrics, Inc. under academic subscription (http://neuromorphometrics.com/).

**Table 1:** **Activated voxels from contrast [erotic > romantic pictures]**.

**Peak Locations**	**Side**	**MNI Coordinates**			**Voxels**	***T***	***p***
		***x***	***y***	***z***			
**EXTENDED CLUSTER 1^a^: TEMPORAL, OCCIPITAL**							
Inferior Occipital Gyrus	L	−45	−73	−2	2149	10.85	0.0001
* Fusiform Gyrus*	L	−42	−49	−17		5.00	< 0.0001
* Superior Occipital Gyrus*	L	−27	−73	31		4.87	< 0.0001
**EXTENDED CLUSTER 2^a^: TEMPORAL, PARIETAL, OCCIPITAL**							
Inferior Occipital Gyrus	R	51	−67	−2	1615	12.43	0.0001
* Inferior Occipital Gyrus*	R	36	−82	10		9.97	0.0001
Middle Occipital Gyrus^*^	R	33	−88	28		6.52	< 0.0001
**FRONTAL LOBE**							
Superior Frontal Gyrus Medial	L	−6	56	16	185	3.68	0.0034
* Superior Frontal Gyrus Medial*	L	−9	50	10		3.64	0.0038
* Superior Frontal Gyrus Medial*	L	−6	50	−5		3.30	0.0109
Superior Frontal Gyrus	L	−24	50	40	60	3.63	0.0040
Superior Frontal Gyrus	L	−18	38	55	31	3.30	0.0108
**OCCIPITAL LOBE**							
Precuneus	R	6	−52	37	40	3.12	0.0182

T-contrast, p < 0.05, FDR-corrected at voxel level, cluster size > 15. Local maxima more than 8 mm apart were extracted and reported. Side indicates hemisphere in which peak voxel is located (R, right; L, left). Labels provided by Neuromorphometrics, Inc. under academic subscription. Entries in italics indicate sub-peak regions within the cluster indicated above.

* Indicates nearest gray matter approximation, Voxels indicates N voxels, T indicates peak t-values, p indicates peak p-values.

a For extended clusters (>1000 Voxels) we extracted and reported local maxima > 20 mm apart in order to illustrate the cluster adequately.

One research objective was to identify brain regions which are modulated both by implicit emotional, as well as explicit task-directed attention. Accordingly, to find voxels displaying main effects that are common to, as well as distinct from *Task* and *Picture Category*, respectively, conjunction plots were created by overlaying both thresholded main effects^4^. Regarding the interactions, significant activations were only found for the *Task*-by-*Picture Category* contrast. To assess whether the according main effects were also qualified by this interaction a further conjunction plot was created overlaying these activation maps with the interaction contrast. Finally, to assess the exact pattern of the interaction in voxels showing main and interaction effects, the averaged beta values across the main clusters of common activation were extracted for each participant and then submitted to repeated-measures ANOVAs.

Reaction time (RT) data provide a behavioral test of response preferences. Error trials and outliers (i.e., trials faster than 300 ms and slower than three standard deviations above the RT mean) were excluded from the RT analyses, resulting in an average of 41 trials per cell. These trials were entered into repeated-measures ANOVA incorporating the factors *Picture Category* (erotic vs. romantic), *Word Category* (positive vs. neutral), and *Task* (picture categorization vs. word categorization). Error rates were very low (*M* = 4.8%) and were not examined further.

## Results

### Reaction times

Participants responded faster to pictures (*M* = 712.1 ms) than to words (*M* = 817.2 ms), *Task*: *F*_(1, 28)_ = 68.2, *p* < 0.001. This main effect, however, was qualified by a *Task* by *Picture Category* interaction, *F*_(1, 28)_ = 29.7, *p* < 0.001. *Post-hoc* tests revealed that participants responded faster to erotic (*M* = 692.7 ms) than to romantic (*M* = 731.5 ms) pictures during the picture categorization task, *t*_(28)_ = 4.4, *p* < 0.001. However, if the participants had to categorize words, erotic (*M* = 823.5 ms) pictures prompted relatively slower responses compared to romantic control images (*M* = 811.0 ms), *t*_(28)_ = 2.3, *p* < 0.05.

### fMRI

#### Emotion main effects

**Figure 2:**
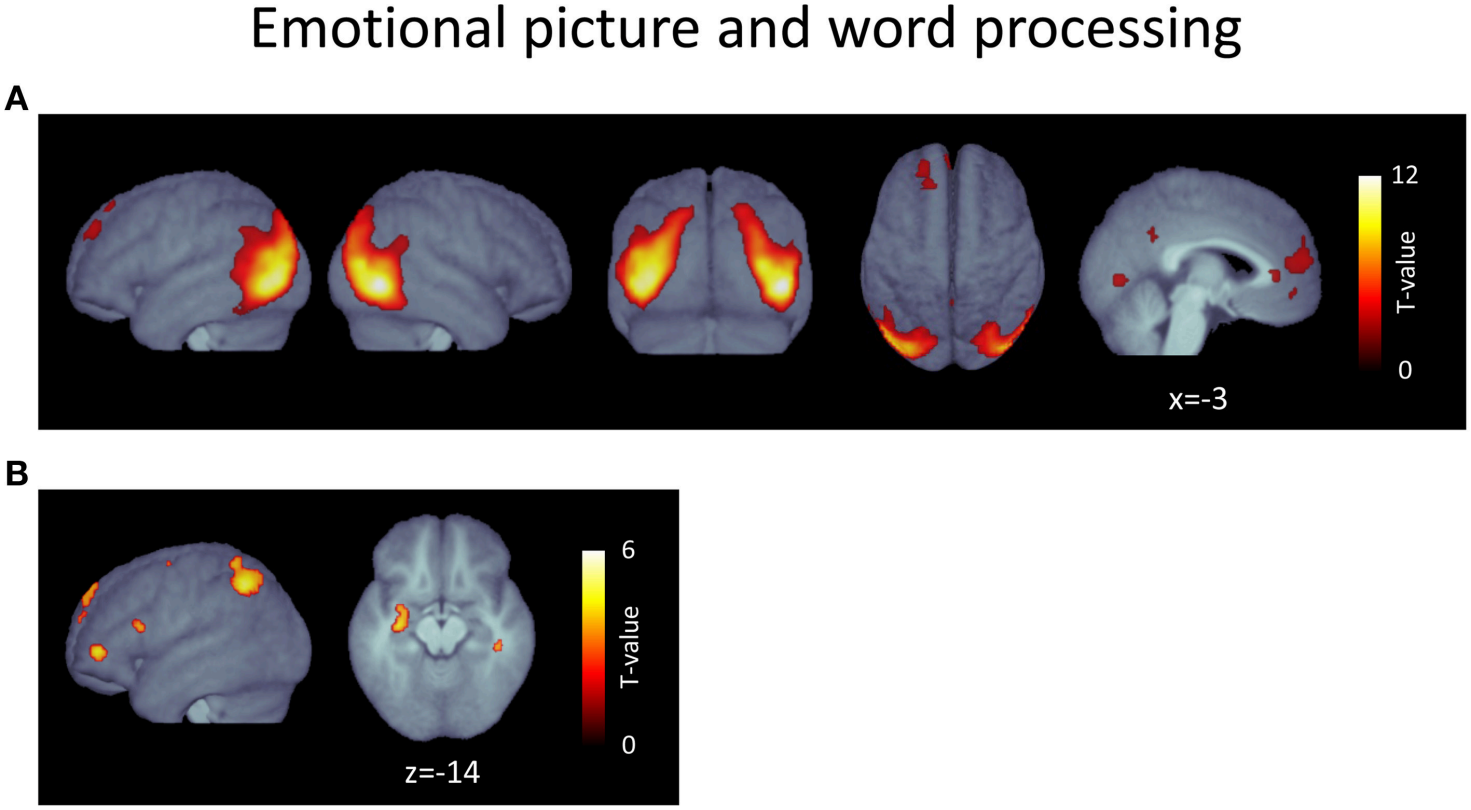
**(A)** Voxels responding more strongly to erotic than to romantic pictures (erotic > romantic). **(B)** Voxels responding more strongly to positive than to neutral words (positive > neutral). *p* < 0.05, FDR-corrected at voxel level; *k* > 15; please note the different scales.

Contrasting erotic with romantic images ([erotic > romantic]) yielded sizeable activations in bilateral extrastriate cortical areas (Figure 2A, Table 1). These clusters covered large portions of lateral occipito-temporal cortex, reaching from fusiform areas ventro-laterally up to superior occipital cortex dorsally. Another large cluster was found in medial prefrontal cortex, almost exclusively in the left hemisphere. This activation included the anterior cingulate cortex as well as regions of the frontal pole. Further clusters were located in the left-sided superior frontal gyrus and in the precuneus.

Contrasting positive with neutral words ([positive > neutral]) predominantly resulted in activation clusters located in the left hemisphere (Figure 2B, Table 2). The largest was found in left parietal regions, mostly covering areas in the vicinity of the intraparietal sulcus and neighboring angular gyrus and reaching into superior parietal lobe. Two further clusters were located in the left inferior frontal gyrus: the larger located in the anterior portion, the smaller more posteriorly. Further clusters were apparent in the left-hemisperic medial superior frontal cortex and posterior superior frontal gyrus as well as in the right cerebellum and temporal lobe. Most notably, a final cluster was found in anterior regions of the left hippocampus, extending into the left amygdala^5^.

**Table 2:** **Activated voxels from contrast [positive > neutral words]**.

**Peak Locations**	**Side**	**MNI Coordinates**			**Voxels**	***T***	***p***
		***x***	***y***	***z***			
**FRONTAL LOBE**							
Superior Frontal Gyrus Medial	L	−6	50	46	42	3.91	0.0235
* Superior Frontal Gyrus Medial*	L	−6	56	37		3.84	0.0273
* Superior Frontal Gyrus Medial*	L	−9	56	25		3.84	0.0273
Middle Frontal Gyrus^*^	L	−39	47	1	37	4.60	0.0071
Superior Frontal Gyrus	L	−24	−7	58	19	3.89	0.0244
Inferior Frontal Gyrus Opercularis^*^	L	−42	17	19	17	3.94	0.0234
**PARIETAL LOBE**							
Angular Gyrus	L	−36	−55	46	241	5.44	0.0018
Superior Parietal Lobule^*^	L	−30	−52	37		4.53	0.0081
* Superior Parietal Lobule*	L	−30	−52	64		4.03	0.0210
**SUB**−**CORTICAL STRUCTURES**							
Hippocampus	L	−33	−16	−14	43	4.56	0.0078
* Amygdala*	L	−27	−7	−17		3.89	0.0248
**CEREBELLUM**							
Cerebellum Exterior	R	12	−85	−35	31	3.99	0.0225
White matter	R	45	−28	−11	22	3.99	0.0225

T-contrast, p < 0.05, FDR-corrected at voxel level, cluster size > 15. Local maxima more than 8 mm apart were extracted and reported. Side indicates hemisphere in which peak voxel is located (R, right; L, left). Labels provided by Neuromorphometrics, Inc. under academic subscription. Entries in italics indicate sub-peak regions within the cluster indicated above.

* Indicates nearest gray matter approximation, Voxels indicates N voxels, T indicates peak t-values, p indicates peak p-values.

#### Task main effects

**Figure 3:**
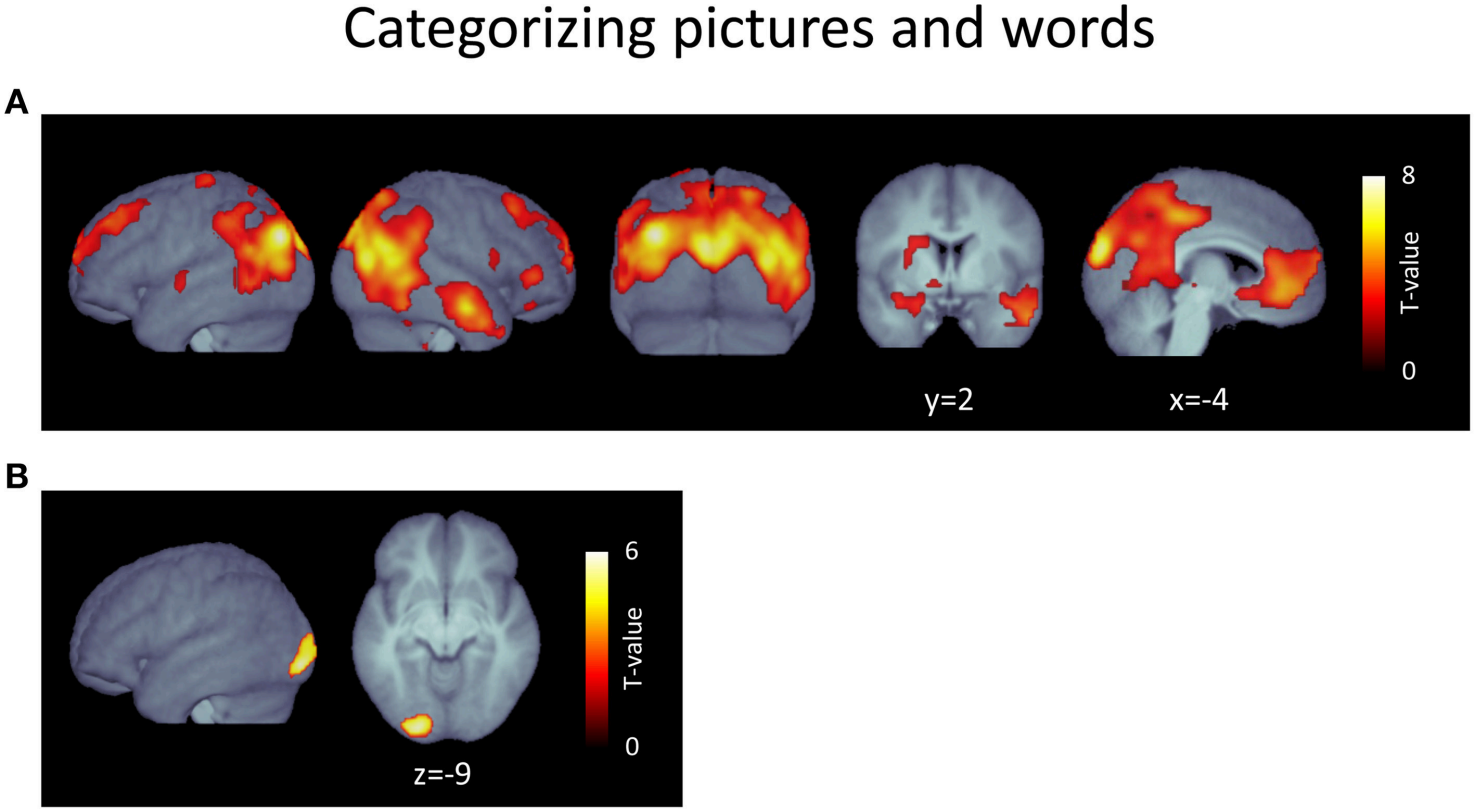
**(A)** Voxels responding more strongly during the picture categorization task (picture > word categorization). **(B)** Voxels responding more strongly during the word categorization task (word > picture categorization). *p* < 0.05, FDR-corrected at voxel level; *k* > 15; please note the different scales.

The contrast [picture categorization > word categorization] resulted in a large contiguous cluster encompassing posterior, frontal, temporal, and subcortical regions (Figure 3A, Table 3). In posterior areas, this included extended activations in bilateral occipito-temporo-parietal regions, reaching into inferior parietal areas and incorporating broad activations in dorso-medial extrastriate regions. It also reached into postero-medial areas covering almost the whole extent of the precuneus and posterior cingulate cortex. Furthermore, this cluster also included strong and sizeable activations of medial regions of the ventral visual stream, including lingual and medial fusiform gyri, parahippocampal areas, and the hippocampus. In frontal regions, this cluster covered large areas of the bilateral medial prefrontal cortex, which included the anterior cingulate cortex and reached into frontal pole regions. Additionally, it extended into left and right lateral prefrontal cortex, including superior and middle frontal gyri. The cluster also included anterior temporal lobe regions exclusively in the right hemisphere, mostly covering middle temporal gyrus, but also reaching into superior and inferior temporal cortex. Finally, subcortical areas were also covered by this extensive cluster. Specifically, this included the posterior thalamus and antero-ventral striatum bilaterally as well as the amygdala, which was activated to a considerably larger extent in the left hemisphere. Further clusters were found in dorsal areas of the left post-central gyrus, in the inferior and orbito-frontal cortex on the right side, and in the left temporal gyrus.

**Table 3:** **Activated voxels from contrast [picture > word categorization]**.

**Peak Locations**	**Side**	**MNI Coordinates**			**Voxels**	***T***	***p***
		***x***	***y***	***z***			
**EXTENDED CLUSTER 1^b^**					**13923**		
**Frontal Lobe**							
* Posterior Cingulate Gyrus*	L	−6	−37	46		5.57	< 0.0001
* Medial Frontal Cortex*	R	3	47	−11		5.42	< 0.0001
* Superior Frontal Gyrus Medial*	L	3	65	19		5.12	< 0.0001
* Middle Frontal Gyrus*	L	−30	44	43		4.25	0.0003
* Middle Frontal Gyrus*	R	24	23	49		4.24	0.0003
* Precentral Gyrus Medial Segment*	R	3	−22	70		2.60	0.0254
Superior Frontal Gyrus^*^	L	−21	59	7		2.43	0.0361
**Temporal Lobe**							
* Temporal Pole*	R	42	17	−38		3.61	0.0021
* Entorhinal Area*	L	−33	2	−20		3.31	0.0047
* Planum Polare*	R	39	−7	−11		3.88	0.0010
**Parietal Lobe**							
Superior Parietal Lobule^*^	R	24	−70	61		4.93	< 0.0001
* Angular Gyrus*	R	54	−46	31		4.67	0.0001
* Precuneus*	R	12	−52	19		4.62	0.0001
* Posterior Cingulate Gyrus*	L	−9	−40	1		4.43	0.0002
* Supramarginal Gyrus*	L	−60	−43	40		3.96	0.0008
**Occipital Lobe**							
* Middle Occipital Gyrus*	L	−39	−82	31		7.96	< 0.0001
* Cuneus*	L	−3	−91	22		7.27	< 0.0001
Fusiform Gyrus^*^	R	30	−46	−14		7.12	< 0.0001
* Middle Occipital Gyrus*	R	39	−82	16		6.64	< 0.0001
* Lingual Gyrus*	L	−24	−52	−11		6.37	< 0.0001
* Inferior Occipital Gyrus*	R	54	−64	13		6.30	< 0.0001
* Superior Occipital Gyrus*	R	24	−85	34		5.53	< 0.0001
* Lingual Gyrus*	R	12	−34	−8		4.83	0.0001
Occipital Fusiform Gyrus^*^	R	33	−76	−8		3.95	0.0008
**Sub-Cortical Structures**							
* Putamen*	L	−33	−13	1		3.50	0.0029
* Caudate*	L	−18	−4	22		3.32	0.0046
Parietal Operculum^*^	L	−33	−22	22		3.09	0.0083
* Ventral Diencephalon*	R	21	−16	−5		2.96	0.0115
* Parahippocampal Gyrus*	L	−21	−13	−26		2.56	0.0273
**Cerebellum**							
* Cerebellum Exterior*	R	21	−40	−47		3.21	0.0061
**FRONTAL LOBE**							
Inferior Frontal Gyrus Triangularis	R	54	41	4	64	3.88	0.0010
Posterior Orbital Gyrus	R	33	38	−17	32	3.18	0.0066
Inferior Frontal Gyrus Opercularis	R	48	11	19	32	2.89	0.0133
**TEMPORAL LOBE**							
Planum Polare^*^	L	−51	−10	−2	35	2.89	0.0135
**PARIETAL LOBE**							
Postcentral Gyrus	L	−21	−31	70	141	3.78	0.0013
Precentral Gyrus^*^	L	−21	−19	61		2.72	0.0198

T-contrast, p < 0.05, FDR-corrected at voxel level, cluster size > 15. Local maxima more than 8 mm apart were extracted and reported. Side indicates hemisphere in which peak voxel is located (R, right, L, left). Labels provided by Neuromorphometrics, Inc. under academic subscription. Entries in italics indicate sub-peak regions within the cluster indicated above.

* Indicates nearest gray matter approximation, Voxels indicates N voxels, T indicates peak t-values, p indicates peak p-values.

b For extended clusters (>1000 Voxels) we extracted and reported local maxima more than 20 mm apart in order to illustrate the cluster adequately. Regions with several sub-peaks were summarized and only the largest peak of the sub-region is reported.

The contrast [word categorization > picture categorization] revealed only a single cluster located in the early extrastriate cortex in the left hemisphere (Figure 3B, Table 4).

**Table 4:** **Activated voxels from contrast [word > picture categorization]**.

**Peak Locations**	**Side**	**MNI Coordinates**			**Voxels**	***T***	***p***
		***x***	***y***	***z***			
**OCCIPITAL LOBE**							
Occipital Fusiform Gyrus	L	−21	−94	−8	229	5.79	0.0005
* Occipital Pole*	L	−21	−100	7		4.63	0.0029

T-contrast, p < 0.05, FDR-corrected at voxel level, cluster size > 15. Local maxima more than 8 mm apart were extracted and reported. Side indicates hemisphere in which peak voxel is located (R, right; L, left), Voxels indicates N voxels, T indicates peak t-values, p indicates peak p-values. Labels provided by Neuromorphometrics, Inc. under academic subscription. Entries in italics indicate sub-peak regions within the cluster indicated above.

#### Overlap of main effects

**Figure 4:**
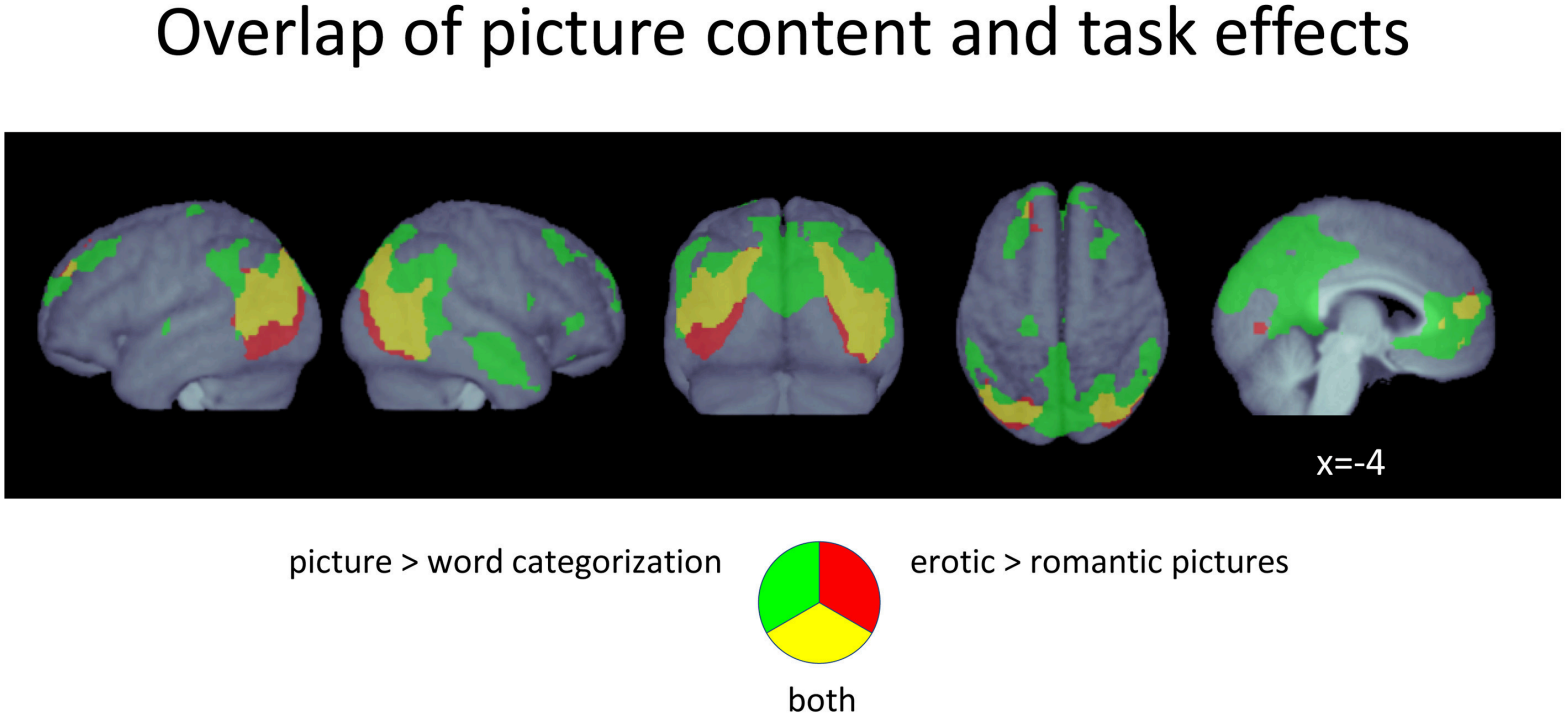
**Conjunction plot of voxels responding both to picture categorization as well as to erotic pictures**. *p* < 0.05, FDR-corrected at voxel level; *k* > 15.

Comparing main effects of picture categorization and picture emotionality showed that the activations found for the processing of erotic pictures were to a large degree also activated when participants had to categorize pictures (Figure 4). Specifically, the vast extra-striate activations for both main effects largely overlapped each other, although they were generally even more extended for the picture categorization contrast. Only relatively few more inferiorly located voxels in the lateral occipito-temporal cortex were exclusive to erotic picture viewing. All additional clusters found for picture emotionality in the cuneus as well as the frontal regions also largely overlapped activity associated with the picture categorization task.

In contrast, main effects of word categorization and word emotionality did not yield any commonly activated voxels, at all.

#### Interactions between picture category, word category, and task

**Figure 5:**
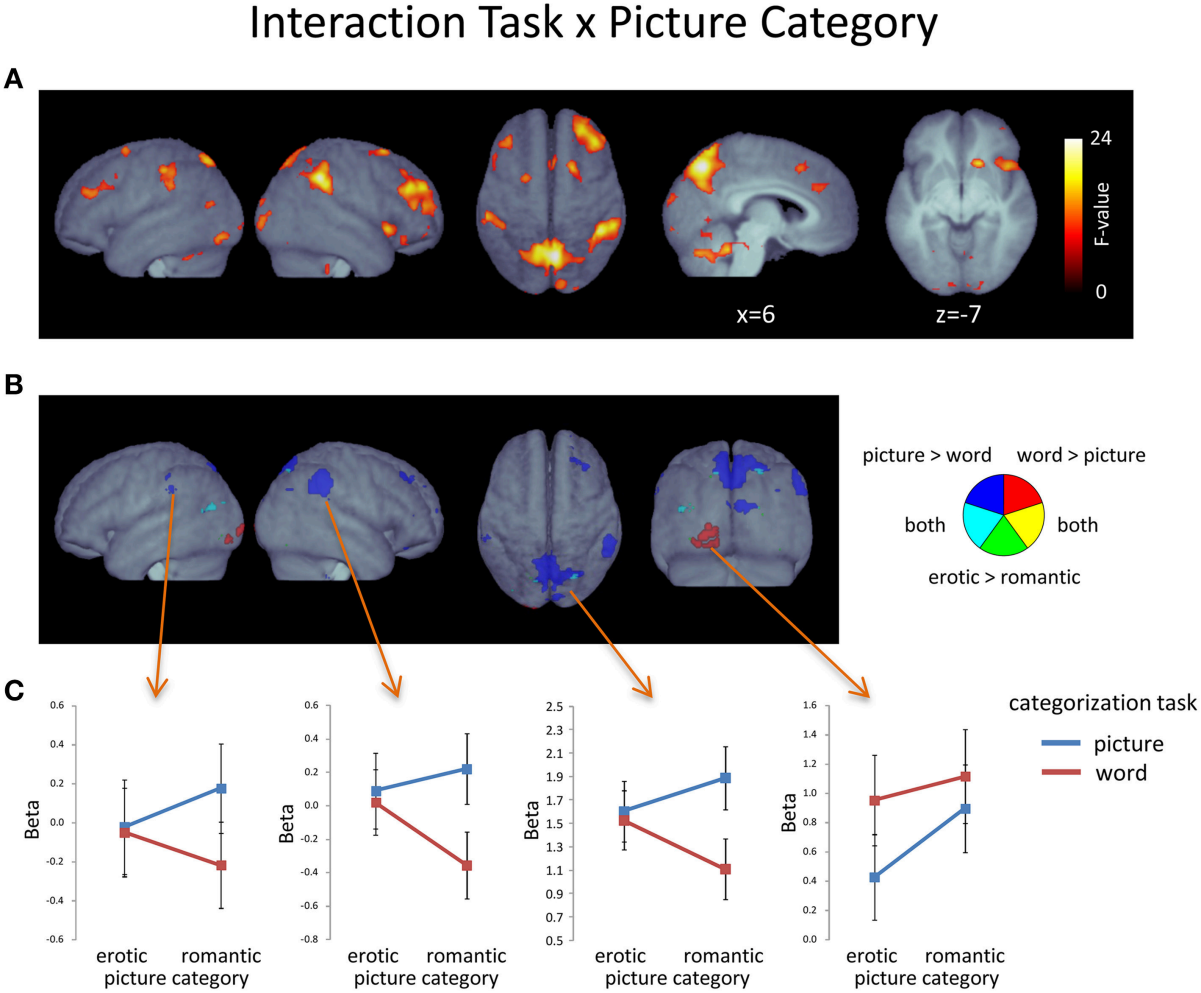
**(A)** Voxels displaying a significant *Task* X *Picture Category* interaction (F-contrast; *p* < 0.05, FDR-corrected at voxel level; *k* > 15). **(B)** Plot of voxels showing the *Task* x *Picture Category* interaction in conjunction with effects of *Task* and/or Picture emotionality. **(C)** Region-of-interest assessment of selected regions showing a conjunction of main and interaction effects. Error bars show standard error of the mean.

As illustrated in Figure 5A (Table 5), a significant interaction between *Task* and *Picture Category* was obtained, consisting of widespread bilateral activations in the dorsolateral-prefrontal cortex, inferior parietal cortex, frontal eye-fields, cerebellum, and the precuneus and cuneus. Further clusters were detected in the right antero-ventral striatum and the right anterior insula, extending into the adjacent inferior frontal cortex and right posterior thalamus. Additional clusters were also found in the pons, pre-SMA, and anterior cingulate cortex. To further detail these findings, we conducted directed interaction T-contrasts for the activated voxels. These confirmed that all voxels were characterized by the same directed interaction pattern. Specifically, in the word categorization task these voxels showed increased activation when the words were overlaid over erotic as compared to romantic pictures. In contrast, this differentiation reversed under the picture task instruction. Here, activity in these voxels was relatively decreased when categorizing erotic as compared to romantic pictures.

**Table 5:** **Activated voxels of ***Task*** x ***Picture Category*** interaction**.

**Peak Locations**	**Side**	**MNI Coordinates**			**Voxels**	***F***	***p***
		***x***	***y***	***z***			
**EXTENDED CLUSTER 1^a^: PARIETAL**							
Superior parietal Lobule	L	−21	−61	43	1227	26.30	0.0049
* Precuneus*	R	6	−70	49		25.80	0.0049
* Superior Parietal Lobule*	R	30	−73	55		12.36	0.0192
* Superior Parietal Lobule*	L	−24	−52	67		9.05	0.0409
**FRONTAL LOBE**							
Middle Frontal Gyrus	R	42	38	34	699	19.39	0.0065
* Middle Frontal Gyrus*	R	30	50	31		18.27	0.0071
Middle Frontal Gyrus^*^	R	27	47	13		17.86	0.0073
Middle Frontal Gyrus	L	−39	38	28	140	14.59	0.0124
* Middle Frontal Gyrus*	L	−54	26	31		12.69	0.0179
* Middle Frontal Gyrus*	L	−51	23	40		11.43	0.0236
Supplementary Motor Cortex	L	3	17	49	62	14.56	0.0125
Superior Frontal Gyrus Medial	R	9	32	28	55	13.15	0.0163
Middle Cingulate Gyrus^*^	R	18	20	34		10.91	0.0264
Superior Frontal Gyrus	R	21	14	67	54	18.40	0.0070
* Middle Frontal Gyrus*	R	30	2	64		9.94	0.0333
Superior Frontal Gyrus	L	−24	5	70	43	14.71	0.0121
* Middle Frontal Gyrus*	L	−27	5	55		9.33	0.0382
Precentral Gyrus	R	39	−10	40	22	13.00	0.0167
* Precentral Gyrus*	R	42	−4	31		11.42	0.0236
Precentral Gyrus	L	−30	−4	40	16	12.54	0.0184
**PARIETAL LOBE**							
Supramarginal Gyrus^*^	R	45	−43	40	701	25.44	0.0049
* Supramarginal Gyrus*	R	57	−40	34		20.04	0.0060
Angular Gyrus^*^	R	36	−55	40		19.13	0.0066
Supramarginal Gyrus^*^	L	−42	−31	37	305	24.91	0.0049
* Supramarginal Gyrus*	L	−42	−40	43		17.38	0.0076
* Supramarginal Gyrus*	L	−60	−31	49		16.89	0.0082
**OCCIPITAL LOBE**							
Superior Occipital Gyrus^*^	R	21	−88	10	675	25.36	0.0049
* Cerebellum WM*	L	−21	−58	−32		22.49	0.0049
Inferior Occipital Gyrus^*^	L	−33	−85	−14		15.72	0.0097
Middle Occipital Gyrus	L	−42	−76	19	59	12.96	0.0168
Angular Gyrus^*^	L	−48	−67	13		10.10	0.0321
Middle Occipital Gyrus^*^	L	−30	−76	13		9.82	0.0344
**SUB-CORTICAL STRUCTURES**							
Caudate^*^	R	15	20	−5	245	19.18	0.0066
Frontal Operculum^*^	R	45	17	−5		18.02	0.0072
* Anterior Insula*	R	33	23	−2		13.42	0.0155
Thalamus Proper	R	24	−22	1	46	21.57	0.0052
Brain Stem	L	−6	−28	−29	40	17.33	0.0076
**CEREBELLUM**							
Cerebellum WM	R	12	−58	−35	644	25.59	0.0049
* Cerebellar Vermal Lobules VI–VII*	R	0	−70	−32		21.61	0.0052
* Cerebellum WM*	R	6	−46	−26		17.59	0.0074
Cerebellum Exterior	R	27	−40	−44	24	12.22	0.0199
Cerebellum Exterior	R	36	−46	−26	23	10.78	0.0273
* Cerebellum Exterior*	R	33	−52	−35		9.85	0.0342

F-contrast, p < 0.05, FDR-corrected at voxel level, cluster size > 15. Local maxima more than 8 mm apart were extracted and reported. Side indicates hemisphere in which peak voxel is located (R, right, L, left). Labels provided by Neuromorphometrics, Inc. under academic subscription. Entries in italics indicate sub-peak regions within the cluster indicated above.

* Indicates nearest gray matter approximation, Voxels indicates N voxels, F indicates peak F-values, p indicates peak p-values).

a For extended clusters (>1000 Voxels) we extracted and reported local maxima more than 20 mm apart in order to illustrate the cluster adequately.

To determine whether the effects of picture emotionality were qualified by this interaction, we compared them with regard to the found interaction pattern. From Figure 5B it becomes apparent that there was no substantial overlap between this interaction and brain regions showing a significant main effect of *Picture Category*, i.e., increased activation to erotic as compared to romantic pictures.

In contrast, the effects of *Task* yielded several regions of overlap with the found interaction (Figure 5B). Most notably, these included large portions of the left-hemispheric extrastriate activations, for the word categorization task, and sizeable regions of the precuneus and both the left and right inferior parietal cortex, for the picture categorization task. Region-of-interest assessment of these voxels (Figure 5C) revealed that precuneus and inferior parietal regions only showed task-related activation differences when participants viewed romantic images. In contrast, the extrastriate region was always more activated during the word, as compared to the picture categorization task—albeit this difference was more pronounced when the words were overlaid onto erotic images.

No significant interactions including the factor *Word Category* were observed.

## Discussion

The present study examined the interplay of implicit emotion and explicit task relevance on the processing of concurrently presented word and picture stimuli. Consistent with the notion of the flexible tuning of processing resources, i.e., benefits of being the focus of attention and cost effects when shared processing resources are taxed, four main findings emerged. First, differential activation of attentional control regions was specific to the picture categorization task, suggesting a pronounced difference between words and pictures in demanding attention regulation. Second, a significant interaction of task and picture category was observed covering large scale neural networks including dorsal visual associative cortex regions and inferior parietal and dorsolateral prefrontal cortices, indicating differential activity to romantic and erotic pictures as a function of task. Third, the selective processing of emotionally arousing pictures and words was independent from task relevance. Fourth, explicit attention enhanced sensory-perceptual processing of pictures and words. Interestingly, only extrastriate activation to words showed effects of competition with picture emotionality as indicated by relatively decreased activity when the words were overlaid over erotic images. Overall, these data suggest the flexible entrainment of large-scale neural networks depending on current behavioral goals and the processing demands of the stimulus, i.e., word or picture and the emotional intensity of the distracter.

### Task effects: words and picture categorization

The present findings suggest a pronounced difference in processing demands associated with the regulation of attention toward pictures and words. Extended activations were observed in corresponding brain regions when the focus of attention was directed toward picture processing. In contrast, none of the neural regions implicated in regulating the allocation of attention to stimuli showed larger activations during the word recognition task. Importantly, these differences were obtained during the processing of stimuli which were physically identical. Furthermore, the task to classify the stimuli was structurally similar for pictures and words, requiring participants to sort the stimuli into two categories defined by emotion. Noteworthily, differences in task difficulty do not seem to account for the pronounced and widespread activations observed for the picture categorization task. Specifically, error rates were low and pictures were classified faster than words, with erotic stimuli showing fastest reaction times. The need to regulate selective attention processes is presumed to depend on demanding task conditions and processing load (Luck et al., 2000; Lavie, 2005). With regard to selectively focus either on the foreground word or background picture, the processing of words showed neither benefits nor cost effects, suggesting little cognitive demand by word processing and indicating automaticity (Augustinova and Ferrand, 2014). In contrast, there was a strong need to regulate processing resources during the picture task, reflecting the flexible tuning of attention processes according to processing goals.

The picture as compared to the word categorization task not only elicited activity in widespread areas of medial and lateral parietal as well as dorso-lateral prefrontal cortices but also in subcortical limbic structures and right temporal areas. While it is difficult to determine whether these effects primarily reflect enhanced activation during the picture task or reduced engagement during the word task, it is clear that the activity in these structures is highly dependent on processing goals. Specifically, the posterior parietal cortex, including the precuneus and lateral parietal areas, has been implicated in visuo-spatial processing, often by using tasks that require visuo-spatial attention shifting (Kastner and Ungerleider, 2000; Simon et al., 2002; Molenberghs et al., 2007; Chica et al., 2013). Additionally, frontal regions in the vicinity of the superior frontal sulcus have also been shown to be involved in voluntary attention shifting and as acting in concert with medial and lateral parietal areas to provide voluntary attentional control in the perceptual as well as the mnemonic domain (Tamber-Rosenau et al., 2011). This conforms well to the present results and it may accordingly be presumed that the processing of pictures invoked attention shifts to a larger degree than word stimuli. From a broader perspective, widespread activity has also been reported for goal-directed stimulus processing and successful recognition memory in neural networks that show a striking overlap to the pattern of findings observed here. For instance, a supramodal limbic-paralimbic-cortical network has been identified by contrasting the processing of Go and NoGo stimuli (Laurens et al., 2005). Furthermore, Keightley et al. (2011) reported regions associated with successful recognition of visual stimuli including ventral prefrontal areas, subcortical structures such as the amygdala and hippocampus, and regions of the anterior temporal lobe which were also restricted to the right hemisphere. Overall, focusing attention on pictures was associated with modulations in cortical and subcortical limbic regions implicated in goal-directed picture processing and recognition memory.

The present findings concur with the notion that selective attention enhances sensory-perceptual stimulus processing. This was apparent regarding the intentional processing of both words as well as pictures. Here, left-lateralized areas of early extrastriate cortex responded most strongly when words were the focus of attention. This result relates to previous reports of visual word processing (Wandell, 2011; Price, 2012) as well as to the present finding of extended bilateral extrastriate activations during picture categorization. This finding, in turn, aligns well with previous studies, suggesting that selective attention to pictures or to specific features of a picture amplifies the perceptual encoding of these features in extrastriate visual cortex (Kastner and Ungerleider, 2000; Pessoa et al., 2002; Jehee et al., 2011). Overall, selective attention to pictures was associated with increased activity in higher-order temporo-occipital visual areas related to object recognition (Grill-Spector and Malach, 2004) while attention to words was reflected in left-lateralized areas devoted to visual word processing.

### Interaction effects: task by picture category

Amplifying the pronounced differences in the engagement of attention-related regions by the goal to process the pictures, interaction effects of task and emotional intensity were only seen for pictures but not words. The posterior parietal cortex and precuneus belonged to the regions in which the main effect of task was further qualified by an interaction with *Picture Category*. Detailed assessment of this interaction revealed that the main effect was largely carried by relative activation increases to romantic pictures in the picture task as compared to the word task, while no differential response was apparent to erotic images (see also Supplementary Figure 1). Previous research has shown that emotional images automatically direct saccades (Calvo and Lang, 2005; Nummenmaa et al., 2009) and facilitate spatial orienting toward these stimuli (Ohman et al., 2001; Koster et al., 2004; De Houwer and Tibboel, 2010). Furthermore, the posterior parietal cortex and precuneus are believed to be important regions involved in the regulation of visuo-spatial attention (Vossel et al., 2014). One hypothesis is accordingly that the interaction observed in these regions reflects that erotic images inherently direct visual attention toward features facilitating recognition and categorization regardless of the task requirements while spatial attention needs to be voluntarily directed toward relevant features when romantic pictures have to be categorized.

A number of regions were observed which revealed interaction effects without overlapping task effects. These included sizeable activations in the bilateral dorso-lateral prefrontal cortex, frontal eye-fields, intra-parietal regions, and midline regions, including pre-SMA, the anterior cingulate cortex, and the right anterior insula. Follow-up analyses characterized the interaction pattern as relatively enhanced activation toward romantic pictures during the picture categorization task and relatively enhanced activation toward erotic pictures during the word categorization task. With regard to the understanding of potentially underlying processes, a previous study by Wessa et al. (2013) appears particularly informative (see also Iordan et al., 2013). Specifically, the authors examined the effects of emotional pictorial distracters on mental arithmetic. Assessing task-execution under the presence of emotional as compared to neutral pictures, they report a strikingly similar pattern of brain networks and emphasize these regions' importance for the upholding of task goals under conditions of emotional distraction. Their experiment directly corresponds to the word categorization task in the present study, in which the picture stimuli are task-irrelevant. Here, the picture stimulus dimension effectively acts as a distracter and this appears to be particularly pronounced for erotic stimuli. However, this may at first seem to be at odds with increased activity toward romantic pictures under the picture categorization task. Conceivably, while acting as distracters during word categorization, erotic pictures may instead facilitate categorization under the picture task instruction. Under this premise, the found interactions likely reveal the differential activation of brain networks involved in maintaining task goals under differential demands for executive control. The reaction time data also corroborate this notion as they indicate a response benefit of erotic pictures in the picture task which apparently translates into a disadvantage in the word task. Additionally, this conclusion is further supported by research utilizing visual Stroop tasks. In related studies, networks largely compatible with the present observations are often implicated in conflict processing (Roberts and Hall, 2008). Interestingly, exclusively right-hemispheric activation of the anterior insula, as observed here, has previously been associated with conflicting approach-withdrawal reaction tendencies brought forward by highly-arousing, positive stimuli (Citron et al., 2014). Finally, the anterior insula has also been suggested to be associated with emotional awareness by integrating bottom-up and top-down information (Gu et al., 2013). This aligns well with the present study in which participants had to cognitively evaluate a stimulus while this stimulus's emotional salience called upon involuntary physiological reactions. The observation that the emotionality of words apparently did not affect task-related activation underscores the pre-eminence of processing pictorial information. In sum, the networks brought forward by the task-by-picture category interaction likely reflect task-related processing which may be facilitated or impeded depending on the emotional intensity of the pictures.

### Stimulus effects: processing of emotional pictures and words

Previous research indicated that the processing of emotional pictures and words is seen in distinct brain regions. The present study confirmed these findings by presenting these two stimulus classes concurrently (see also Kensinger and Schacter, 2006). With regard to pictures, the processing of high-arousal erotic as compared to low-arousal control pictures was associated with increased activations in extended regions of the extrastriate visual and inferior temporal cortices. Previous research observed that the sensory-perceptual processing of emotional stimuli varies with the availability of processing resources (Pessoa et al., 2002; De Cesarei et al., 2009; Schupp et al., 2014). However, given the strong and sizeable effects observed both for the interaction between task and picture category, as well as for erotic picture viewing, modulations of the latter by task focus seen in visual processing regions were comparably minute. This presumably reflects little competition by words for processing resources claimed by erotic pictures. Given that no interaction with word category was found, this attests to a strong attentional bias toward erotic pictures and highlights the automaticity and expertise in extracting semantic meaning from pictures and words (Thorpe et al., 1996; Augustinova and Ferrand, 2014). Furthermore, larger activations in regions of the dorso-medial prefrontal cortex and the precuneus using erotic stimuli replicated previous research investigating emotional stimulus processing (Sabatinelli et al., 2011; Lindquist et al., 2012). However, the present study did not observe a differential response to the picture categories in sub-cortical limbic structures, most notably the amygdala, which has often been observed to be associated with erotic stimulus processing. The difference in findings may relate to the control category. Specifically, the picture control category depicted couples in pleasant romantic contexts, and the affective distance between the stimulus categories may have been sub-optimal in bringing forward emotional differentiation in the amygdala and other limbic regions. This interpretation possibly relates to findings that these regions respond to both highly and mildly arousing social stimuli (Goossens et al., 2009; Vrticka et al., 2013). This reasoning is also broadly consistent with the observation in the present study that the amygdala was activated when attention was explicitly directed toward pictures, regardless of picture category. This may be taken as an indication for competition between explicit task demands and implicit attention in the amygdala (Pessoa et al., 2003; Hsu and Pessoa, 2007).

The processing of positive as compared to neutral words led to increased activations in several left-lateralized clusters, including the inferior and medial superior frontal gyri, left parietal cortex, left hippocampus, and amygdala. These findings largely replicate strongly left-lateralized activation patterns reported in previous studies of emotional word processing (Kensinger and Schacter, 2006; Herbert et al., 2009; Hoffmann et al., 2015) and are consistent with the view of left-lateralized language functions in humans (Price, 2012). More specifically, areas in left ventrolateral prefrontal, mesial superior frontal and inferior parietal regions have all been connected to semantic and evaluative processing of language (Devlin et al., 2003; Salmelin and Kujala, 2006; Binder et al., 2009; Price, 2012). Interestingly, in the present study the finding of enhanced activations in extrastriate visual cortex associated with the processing of words depended on task focus and the goal-directed allocation of attention. Specifically, although cortical brain regions related to semantic stimulus processing and limbic regions related to affective evaluation responded to word emotionality irrespective of task, increased activations in extrastriate regions to words were only seen when participants were conducting the word categorization task. This observation relates to a recent study examining neural correlates of reading (Hillen et al., 2013). In this study, activation in according extrastriate regions was associated with the visual scanning of written language but not with semantic, syntactic, or orthographic processing. These processes in contrast were most notably associated with activation in areas of left-lateralized prefrontal cortex. This study's results are highly reminiscent of the present observations regarding word processing and suggest a dissociation of sensory-perceptual and affective-semantic processing in extrastriate and prefrontal/subcortical regions, respectively. While affective-semantic evaluation of the words seems to be automatic and undisturbed by task demands or picture emotionality, perceptual processing of words during reading is affected by both processes as indicated by the interaction in extrastriate cortex (Figure 5C). In addition, considering that other research reported similar activations to words also during cognitively undemanding silent reading (Herbert et al., 2009), extrastriate activity to visually presented words may thus not depend on task focus *per se*. Rather, these observations are consistent with the view of competition for shared resources in extra-striate visual cortex while activity in stimulus-specific semantic and limbic word processing regions is preserved (Lavie, 2005). One may accordingly speculate that the increased activation in extra-striate cortex reflects recurrent processing loops flexibly engaged depending on behavioral goals and the availability of processing resources. Overall, regarding emotional word processing the present data suggest a dissociation of semantic and affective evaluative processes, on the one hand, and sensory processing, on the other hand, when explicit attention is directed toward pictures.

### Limitations

While the present design was successful at detailing common and specific brain responses to the implicit emotional significance of pictures and words as well as to explicit attentional demands, some characteristics of the used stimuli require further consideration. Specifically, the emphasis on stimulus selection was on the emotional arousal dimension and the comparability of the stimulus categories in terms of linguistic parameters of the words, i.e., word length, number of syllables, imageability, and word frequency as well as stimulus characteristics of the image, i.e., picture complexity, color, number of people and categorical homogeneity. High control on some stimulus properties led to differences in other characteristics. Specifically, pictures were drawn from selected categories of human experience while words represented a broad range of experiences. Furthermore, while both stimulus classes differed in emotional arousal, the strong physical and semantic control exerted for the pictures made it not feasible to select a control category differing both in arousal, as well as valence. Thus, while emotional modulation of word processing may be attributed either to variations in arousal or valence, differentiations due to picture category may only be associated with arousal. This may account in part for the lack of congruency and/or incongruency effects between picture and word categories in the present results (Klasen et al., 2011). In addition, extensive previous research has demonstrated that the preferential processing of emotional stimuli is associated both with common, but also with distinct brain regions depending on emotional valence and arousal, as well as specific emotional content (Vytal and Hamann, 2010; Sabatinelli et al., 2011; Citron, 2012). With regard to erotic pictures, the regions found in the present study are not characterized by high content specificity (Sabatinelli et al., 2011) and thus likely reflect attentional processes evoked by a large variety of emotionally arousing pictures. Regarding words, previous research has detailed differentiations according to valence and arousal of the stimulus materials but also according to whether the emotional connotation of the words had to be processed directly (reviewed in Citron, 2012). However, only one study addressed both issues utilizing fMRI (Straube et al., 2011). Most notably, in this study none of the regions reported here were found to be modulated by task or by stimulus valence. Another study by Citron et al. (2014) orthogonally manipulated both arousal, as well as valence of words using an indirect lexical decision task. Of note, in this study none of the regions reported here were modulated by valence. In addition, several previous studies reported comparable left-lateralized semantic and subcortical limbic regions associated with the processing of both positive, as well as negative emotional words as observed here (Hamann and Mao, 2002; Cato et al., 2004; Kensinger and Schacter, 2006; Herbert et al., 2009; Straube et al., 2011). Thus, the present results most likely reflect selective processing associated with the emotional arousal of the words. However, the present study is not conclusive toward this end and future research should strive to further detail the involvement of specific brain regions in the processing of valence, arousal and emotional task by selecting experimental stimuli which systematically vary with regard to semantic categories, valence (including negative stimuli), and arousal (including low and high arousing stimuli) of the word and picture stimuli.

## Conclusion

The present study examined costs and benefits of the processing of emotionally arousing pictures and words when the stimuli were either task-relevant or task-irrelevant. The implicit significance of emotional stimuli was reflected in distinct brain regions for the processing of pictures and words, respectively. Of note, the activity in these regions was similar when the stimuli were task-relevant or irrelevant suggesting that there was no competition for processing resources in respective brain regions. However, effects of competition were observed in the left-lateralized visual cortex between explicit attention to words and implicit attention to picture emotionality. Finally, widespread fronto-parietal networks were apparent as a function of the interaction between explicit task demands and picture category, specifically. Overall, these results attest to the brain's ability to process emotional information from different visual sources in parallel when these do not share common resources and suggest the flexible entrainment of large-scale neural networks depending on processing goals, obligatory processing demands of the stimulus type, and the emotional intensity of distracter stimuli.

### Conflict of interest statement

The authors declare that the research was conducted in the absence of any commercial or financial relationships that could be construed as a potential conflict of interest.

## Footnotes

^1^Here, the terms “explicit” and “implicit” are used to refer to selective attention processes which need not be specifically aimed at the emotional connotation of a stimulus. Accordingly, they should not be confused with the direct vs. indirect processing of the emotional connotation of a stimulus, for which the same labels are often used in the domain of emotion research.

^2^Full List of word stimuli. Positive: Liebe, Sex, Sonne, Urlaub, Herz, Sieg, Held, Party, Freude, Schatz, Feier, Sommer, Charme, Spaß, Erfolg, Erotik, Gewinn, Gefühl, Lust, Ferien, Glück, Chance; Neutral: Boden, Uhr, Fahne, Neubau, Rede, Form, Test, Besen, Gegend, Treppe, Kabel, Karton, Lesung, Wand, Stelle, Ordner, Urteil, Metall, Note, Klinke, Meter, Inhalt. English translation in same order. Positive: Love, Sex, Sun, Holiday, Heart, Victory, Hero, Party, Joy, Treasure, Celebration, Summer, Charm, Fun, Success, Erotic, Prize, Feeling, Lust, Vacation, Luck, Chance; Neutral: Floor, Clock, Flag, Reconstruction, Talk, Form, Test, Broom, Area, Stairs, Cable, Box, Reading, Wall, Place, Folder, Opinion, Metal, Note, Handle, Meter, Content.

^3^To promote comparability between valence and arousal ratings of pictures and words, respectively, the reported values for words were transformed to a 9-point-Likert scale as utilized for the SAM.

^4^No activations were found common to word categorization and word emotionality. Accordingly, only the conjunction of the main effects for picture categorization and picture emotionality is presented in Figure 4.

^5^For the pictures, the reversed contrast revealed a single cluster of increased activity to romantic, as compared to erotic pictures ([romantic > erotic]). This cluster was located in early visual cortex bilaterally, mainly including occipital pole regions but also extending into cuneus and calcarine cortex (Supplementary Figure 2). In contrast, no further activations were found when comparing neutral with positive words ([neutral > positive]).
