# Supplementary figures and images for: Implicit and Explicit Attention to Pictures and Words: An fMRI-Study of Concurrent Emotional Stimulus Processing
Source: Front Psychol. 2015 Dec 18;6:1861. doi: 10.3389/fpsyg.2015.01861 (PMC4683193; doi:10.3389/fpsyg.2015.01861)

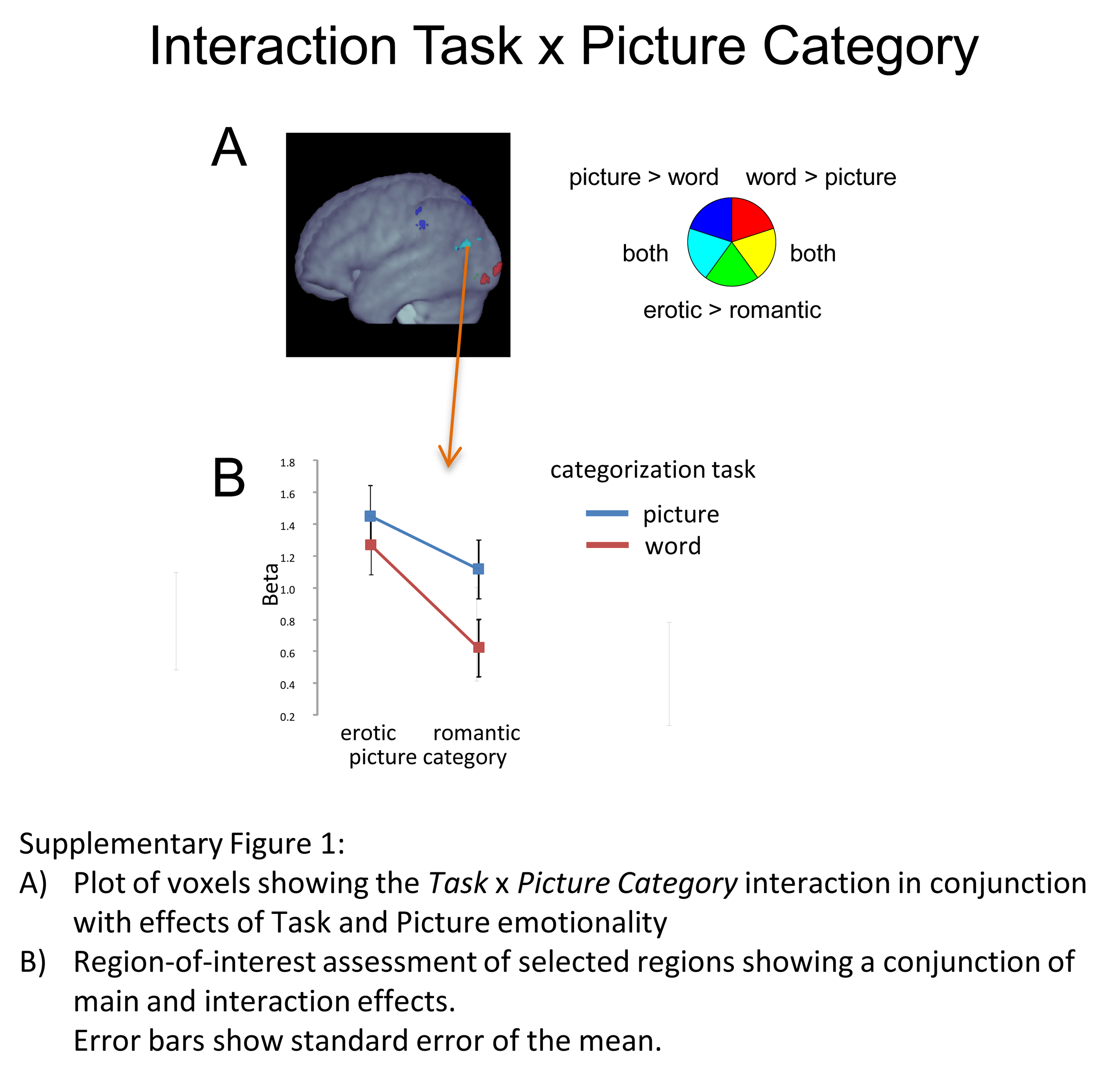

Supplement: Supplementary file 1 [file Image1.TIF]

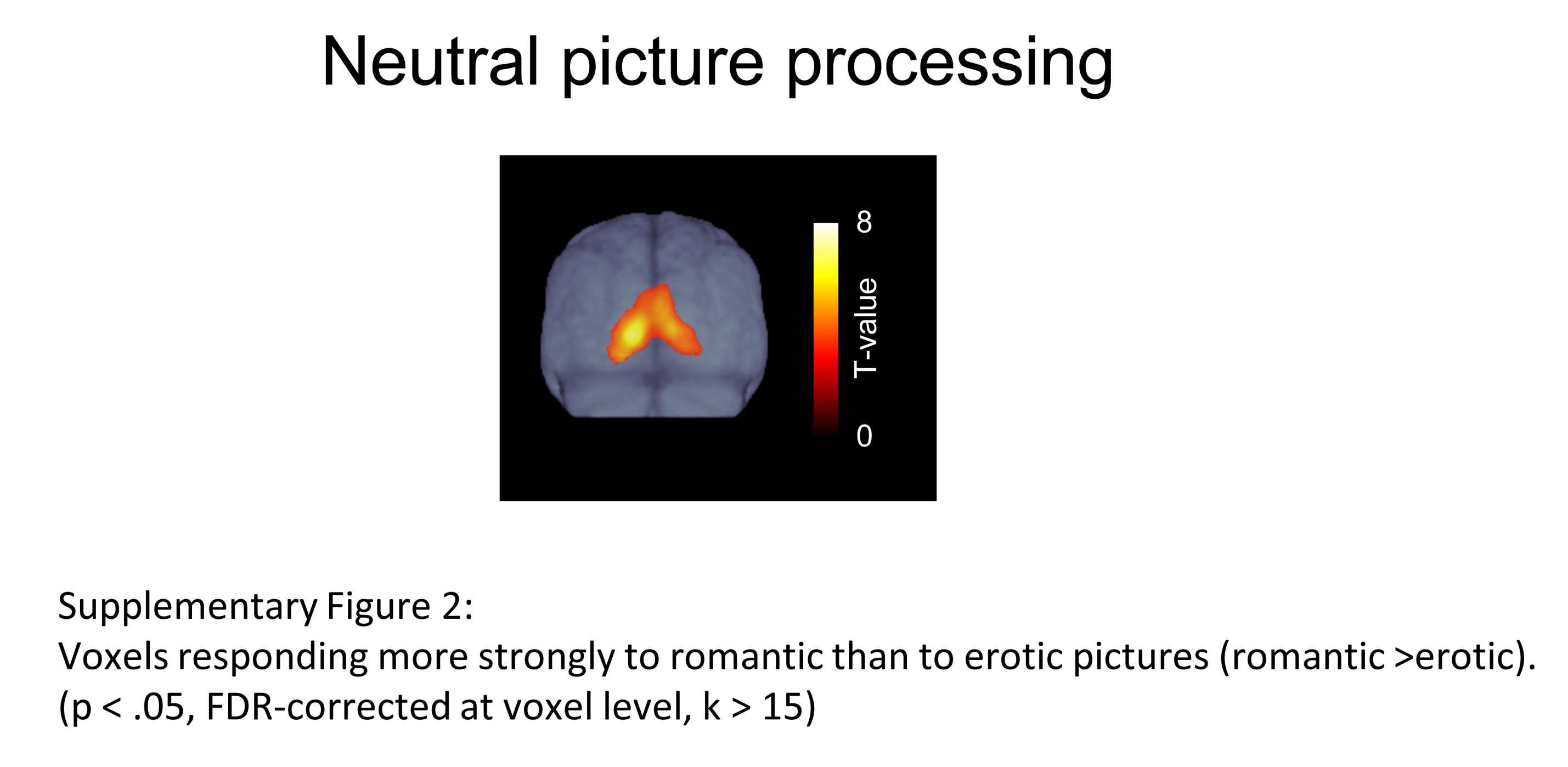

Supplement: Supplementary file 2 [file Image2.TIF]
